# Rotigotine Attenuates Mechanical Hypersensitivity in 6-OHDA-Lesioned Rats: Associations with D2/D3 Receptors and Spinal Glial NF-κB/p65 Signaling

**DOI:** 10.1007/s11064-026-04948-8

**Published:** 2026-11-04

**Authors:** Jazmin Santamaria-Anzures, Guadalupe Martínez-Lorenzana, Estefanía González-Morales, Dafne Palmeros-Barreda, Ledy De-la-Cruz-Martínez, Francisco Cortés-Benítez, Julia Pérez-Ramos, Miguel Condés-Lara, Beatriz Godínez-Chaparro

**Affiliations:** 1 Departamento de Sistemas Biológicos, División de Ciencias Biológicas y de la Salud, Universidad Autónoma Metropolitana-Xochimilco, Calzada del Hueso 1100, Colonia Villa Quietud, Ciudad de México, 04960 México; 2 Departamento de Neurobiología del Desarrollo y Neurofisiología, Instituto de Neurobiología, Universidad Nacional Autónoma de México, Campus UNAM Juriquilla, Querétaro, 76230 México

**Keywords:** Rotigotine, Parkinson’s Disease Pain, Allodynia, Hyperalgesia, NF-κB Signaling, Microglia, Astrocytes, Spinal Cord, 6-OHDA-Lesioned Rats, Dopamine Receptor

## Abstract

Pain is a debilitating non-motor symptom of Parkinson’s disease (PD). This study investigated whether rotigotine attenuated pain hypersensitivity (allodynia and hyperalgesia) in hemiparkinsonian rats and explored D1/D2/D3 receptor involvement, NF-κB/p-p65 signaling modulation in glial cells, and spinal TNF-α and IL-1β expression. The PD model was induced by unilateral 6-OHDA injection into the substantia nigra pars compacta (SNpc). Pain sensitivity was assessed using von Frey filaments. Immunohistochemistry and immunofluorescence were used to analyze tyrosine hydroxylase levels in the SNpc and striatum, GFAP, Iba-1, and NF-κB/p-p65 expression in the dorsal horn of the L5 spinal cord, and ELISA was used to determine TNF-α and IL-1β levels in the lumbar spinal cord. Both acute (8 h) and subacute (5-day) intranasal rotigotine (0.1, 0.5, 0.75, and 1 mg/kg) showed antiallodynic and antihyperalgesic effects. These effects were prevented by selective D2 (L-741,626; 14.6 nmol) and D3 (nafadotride; 3 nmol) receptor antagonists, but not by the D1 receptor antagonist SCH-23,390 (30 nmol). Subacute rotigotine was associated with reduced 6-OHDA-induced glial activation, decreased NF-κB/p-p65 expression, and attenuated spinal TNF-α and IL-1β levels. Molecular docking suggests a potential interaction between rotigotine and the p65 subunit of NF-κB, with favorable binding energy. In conclusion, rotigotine moderated PD-related pain, potentially through D2/D3 receptor activation and modulation of neuroinflammation. This suggested antinociceptive effect may be linked to suppressing glial activation and inhibiting spinal astrocytes’ NF-κB/p-p65 signaling, potentially reducing subsequent proinflammatory cytokine release. These findings suggest that rotigotine may represent a promising therapeutic option for managing sensory hypersensitivity in PD.

## Introduction

Pain is one of the most common and disabling non-motor symptoms of Parkinson’s disease (PD), affecting approximately 70–83% of patients [1]. Multiple pain phenotypes, including nociceptive, neuropathic, and nociplastic pain, have been described in PD [2]. Despite its high prevalence and major impact on quality of life, pain in PD remains underrecognized and inadequately treated. A better understanding of the mechanisms that drive pain hypersensitivity in PD is therefore needed to identify more effective therapeutic strategies.

Accumulating evidence indicates that PD promotes glial overactivation and neuroinflammatory responses characterized by the release of pro-inflammatory mediators such as tumor necrosis factor-alpha (TNF-α) and interleukin-1 beta (IL-1β), which contribute to the induction and maintenance of pain hypersensitivity, including allodynia and hyperalgesia [3]. Consistent with this view, astrocytes and microglia are activated in the spinal cord of PD animal models, including 6-hydroxydopamine (6-OHDA) lesioned mice and rats [4, 5], as well as 1-methyl-4-phenyl-1,2,3,6-tetrahydropyridine (MPTP) treated mice [6]. Glial activation can enhance central nervous system excitability through mechanisms related to central sensitization and long-term potentiation [7]. In this context, nuclear Factor kappa B (NF-κB) signaling is particularly relevant because it regulates the transcription of inflammatory cytokines and other mediators involved in persistent pain. Notably, dopaminergic drugs such as levodopa and pramipexole have been reported to suppress glial activation in the striatum [8] and the spinal cord [5], supporting the idea that inhibiting glial activation may be a useful therapeutic strategy for PD-related pain.

Rotigotine (Ro) is a dopamine agonist with high affinity for D3, D2, D1, D4, and D5 receptors [9] and has been shown to reduce pain hypersensitivity in patients with PD [10] as well as in experimental pain models [11, 12]. However, the mechanisms underlying their analgesic effects remain poorly understood. It is still unclear which dopamine receptor subtypes mediate the antinociceptive actions of rotigotine in PD-related pain. Clarifying this issue is important because it may distinguish rotigotine mechanistically from other dopamine agonists and help explain its therapeutic profile.

Recent studies have shown that rotigotine-loaded microspheres produce antinociceptive effects in inflammatory pain models [11, 12] and these effects are mediated primarily through D2/D3 receptor activation [12]. In parallel, D2 receptor signaling has been reported to suppress inflammation by inhibiting NF-κB activation through a Protein Kinase B (Akt)/ Protein Phosphatase 2 A (PP2A) dependent pathway [13], and rotigotine has also been shown to reduce acute liver injury through regulation of NF-κB signaling [14]. Because NF-κB is a central transcriptional regulator of immune responses and pathological pain [15], it is plausible that rotigotine may alleviate PD-related pain by modulating this pathway in spinal glial cells. Accordingly, this study investigated whether rotigotine attenuates tactile allodynia and mechanical hyperalgesia in 6-OHDA-lesioned rats by activating D1, D2, or D3 receptors and whether these effects are associated with reduced spinal neuroinflammation and inhibition of NF-κB signaling in glial cells.

## Materials and Methods

### Animals

All experimental procedures were conducted in accordance with the Mexican Official Standard for the care and use of laboratory animals (NOM-062-ZOO-1999) and the National Institutes of Health (NIH) Guide for the Care and Use of Laboratory Animals. The Animal Production and Testing Committee (Protocol 251) and the Research Ethics Committee (Protocol 136) of UAM-Xochimilco approved the study design and protocols.

A total of 106 male Wistar rats (280–320 g) were obtained from the Animal Production and Experimentation Unit (UPEAL) of the UAM-Xochimilco, Mexico City. Animals were housed under standard conditions (22 ± 2 °C, 60% humidity) on a 12 h light/dark cycle with free access to food and water. Animal welfare was monitored twice daily. Pre-established humane endpoints include body weight loss exceeding 20% from baseline, severe akinesia, or persistent loss of the righting reflex. Animals meeting these criteria were prematurely and humanely euthanized. At the end of the experiments, all remaining animals were euthanized via gradual filling of CO_2_ in an airtight chamber (displacement rate of 30% of the chamber volume per minute.

### Drugs

6-OHDA (Cat. No. 28094-15-7, Sigma-Aldrich, St. Louis, MO, USA) and apomorphine (Cat. No. 253341 A, ChemCruz, Santa Cruz Biotechnology) were dissolved in 0.9% saline supplemented with 0.02% ascorbic acid (Cat. No. 50-81-7, Sigma-Aldrich, St. Louis, MO, USA). Rotigotine (Cat. No. Y0002005); SCH-23,390 (Cat. No. 12941-87-9, Sigma-Aldrich, St. Louis, MO, USA) was dissolved in 0.9% saline. Nafadotride (Cat. No. 1347, Tocris Bioscience, Bristol, UK) and L-741,626 (Cat. No. 81226-60-0, Sigma-Aldrich, St. Louis, MO, USA) were dissolved in saline containing 0.5% dimethyl sulfoxide (DMSO).

### Stereotaxic Surgery

Rats were anesthetized with ketamine (48 mg/kg) and xylazine (12 mg/kg) administered intraperitoneally (i.p.) and placed in a stereotaxic frame (Stoelting, Wood Dale, IL, USA) in the prone position. After exposing the skull, bregma was used as a reference to locate the injection site at the following coordinates: anteroposterior (AP), − 4.0 mm; lateral (L), + 1.2 mm; and ventral (V), − 8.0 mm [5]. These coordinates were used to inject 6-OHDA or vehicle into the left SNpc. Using a 5 µL Hamilton syringe (Hamilton, Reno, NV, USA), 2 µL of vehicle or 2 µL of 6-OHDA (8 µg/µL; total dose, 16 µg) were injected at a rate of 0.1 µL/min with a Stoelting Quintessential Stereotaxic Injector (QSI). Sham-lesioned rats received the same volume of vehicle. The needle was left in place for 10 min to allow diffusion and then slowly withdrawn. The incision was sutured with 3 − 0 chromic catgut, and an antiseptic solution was applied. To prevent hypothermia, rats were placed on a warming pad until recovery from anesthesia.

#### Behavioral Testing is Used to Assess Motor Performance

**Fig. 1:**
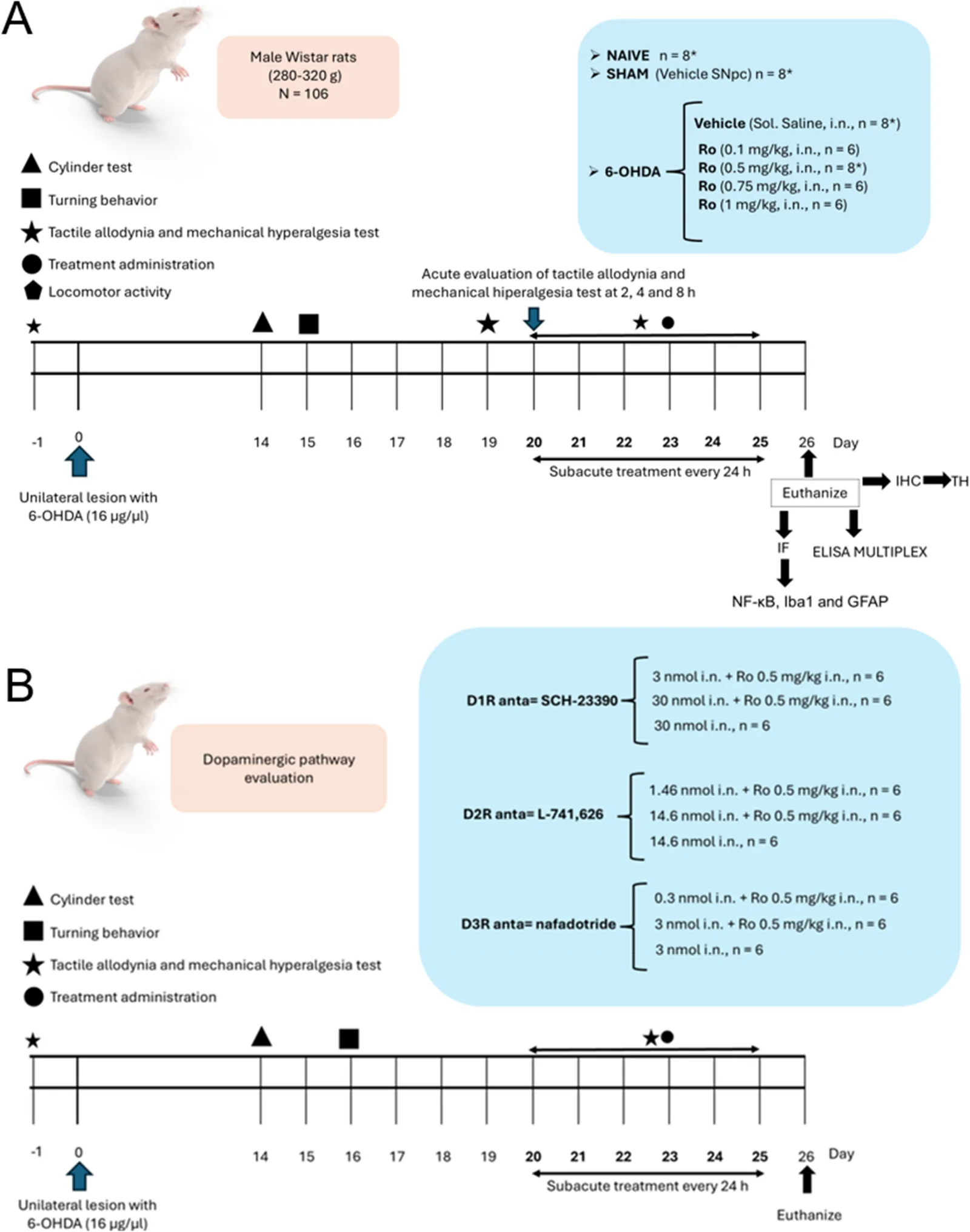
Timeline for experimental design. Rats received a unilateral injection of 2 µL of 6-OHDA at 8 µg/µL (total dose, 16 µg) or vehicle (0.02% ascorbic acid) into the left SNpc. We validated motor deficits using the cylinder test and turning behavior at 14- and 15-days post-lesion, respectively. Sensory assessment of tactile allodynia and mechanical hyperalgesia began 20 days post-surgery and continued until 25 days post-surgery. **A** Acute and subacute (5-day) treatments: rats received intranasal (i.n.) rotigotine (0.1, 0.5, 0.75, and 1 mg/kg/day; *n* = 6, per group) or vehicle. Nociceptive thresholds were evaluated acutely (0, 2, 4, 6, and 8 h post-treatment) and sub-acutely (daily for 5 days). **B** Mechanistic study: to elucidate the dopaminergic receptors underlying the antiallodynic and antihyperalgesic effects of rotigotine, selective antagonists were administered intranasally once daily for 5 days, 10 min before each effective dose of rotigotine (0.5 mg/kg, i.n.): D1 receptor antagonist SCH-23,390 (3 and 30 nmol), D2 receptor antagonist L-741,626 (1.46 and 14.6 nmol), or D3 receptor antagonist nafadotride (0.3 and 3 nmol). Pain-related behaviors were assessed daily before each administration. On day 26, animals were euthanized for subsequent tissue analysis. For immunohistochemistry (IHC) for tyrosine hydroxylase (TH) and immunofluorescence (IF) for ionized calcium-binding adaptor molecule 1 (Iba1), glial fibrillary acidic protein (GFAP), and nuclear factor kappa B p65 (NF-κB/p65), animals underwent transcardial perfusion. Additionally, tumor necrosis factor-alpha (TNF-α) and interleukin-1 beta (IL-1β) levels were quantified using enzyme-linked immunosorbent assay (ELISA) in fresh-frozen tissue samples. *4 rats were used for immunohistochemistry, and 4 rats were used for immunofluorescence

The same experimenter performed the behavioral tests (cylinder test, turning behavior, and open-field test) between 11:00 and 14:00 during the light phase. (Fig. 1) shows the experimental timeline. All sessions were video recorded for subsequent analysis.

### Cylinder Test

Rats were placed in a Plexiglas cylinder (20 cm diameter × 30 cm height), and their activity was video recorded for 5 min. During vertical exploration, forelimb use for wall contact was classified as contralateral forelimb (CL), ipsilateral forelimb (IL), or both forelimbs simultaneously (BF). The number of wall contacts made with each forelimb condition was counted. The test was considered valid when at least eight wall contacts were observed per rat [16].

### Turning Behavior Test Induced with Apomorphine

Animals were placed in a Plexiglas cylinder (20 cm diameter × 30 cm height) and allowed to habituate for 5 min. Apomorphine (0.1 mg/kg, s.c.) was then administered, and behavior was recorded for 30 min. The number of full contralateral turns (360°) relative to the lesion side was counted. Animals exhibiting at least 180 contralateral rotations were included in the study [17].

### Open Field Test

Animals were placed in an acrylic box (44 × 33.5 × 20 cm) divided into 12 squares of 11 cm x 11 cm. Each rat was positioned in the center of the arena and allowed to explore freely for 5 min. The surface was cleaned with 70% ethanol before each trial to eliminate olfactory cues. Locomotor activity was quantified as the number of sectors visited, with each square considered one sector.

### Measurement of Tactile Allodynia

Hind paw withdrawal threshold (PWT) was assessed using von Frey filaments (Stoelting, Wood Dale, IL, USA) to evaluate tactile allodynia. Rats were placed individually in Plexiglas chambers (26 × 20 × 14 cm) on a wire-mesh floor and allowed to acclimate for 20 min before testing. To minimize inter-observer variability, the same investigator performed all behavioral assessments across all experiments.

The PWT was determined using the up-down method described by Dixon and adapted by Chaplan et al. [18]. A series of consecutive von Frey filaments with logarithmically incremental stiffness (0.4, 0.7, 1.2, 2.0, 3.6, 5.5, 8.5, and 15 g) were applied at 5 min intervals. A positive withdrawal response was defined as an abrupt paw lifting or flinching upon filament application. If no response occurred, the next filament with a higher force was applied. If a positive response occurred, the next lower force filament was selected. A force of 15 g was set as the cut-off value to prevent tissue damage; a negative response at this maximum force was recorded as a 15 g threshold.

The 50% withdrawal threshold was calculated using the formula 10(Xf + kδ)/10,000, where Xf is the final von Frey value (log units), k is the tabular value corresponding to the pattern of the last six positive/negative responses, and δ is the mean difference (log units) between stimuli. Tactile allodynia was defined as a PWT below 4 g. Animals exhibiting a baseline withdrawal threshold below 4 g or showing any sign of motor deficiency were excluded from the study.

### Measurement of Mechanical Hyperalgesia

Mechanical hyperalgesia was assessed using a fixed-intensity method with a von Frey filament (Stoelting Co., Wood Dale, IL, USA) delivering a fixed force of 26 g (250 mN) to the plantar surface of each hind paw. The filament was applied 10 times per paw, and each animal underwent three trials to obtain the mean number of withdrawal responses [19]. This stimulus of 250 mN or greater was considered noxious. One or two withdrawal responses were considered within the normal range, whereas values ≥ 5 withdrawal responses were considered indicative of hyperalgesia.

### Perfusion and Tissue Fixation

Rats were anesthetized with isoflurane (3%) and transcardially perfused with 4% paraformaldehyde in 0.1 M phosphate-buffered saline (PBS). Brains and L4–L6 spinal cord segments were removed and post-fixed in paraformaldehyde for 30 min, followed by cryoprotection in 30% sucrose at 4 °C. Brains were coronally sectioned at 60 μm through the striatum and substantia nigra using a freezing microtome (Leica CM1100; Leica Instruments GmbH, Nussloch, Germany) at − 22 °C. Sections were stored in PBS at 4 °C until immunohistochemical processing. L4–L6 spinal cord segments were frozen and coronally sectioned at 40 μm using a freezing microtome (Leica SM2000 R; Leica Instruments GmbH, Nussloch, Germany). Sections were collected in five series, and the first series was processed for immunofluorescence.

### Histological, Immunohistochemical, and Immunofluorescence Analyses

Immunohistochemistry was performed as previously described by Mendieta et al., with minor modifications [20]. Brain sections were washed in 0.4% Triton X-100 in PBS (PBS-T) and incubated in 3% H_2_O_2_ (Fermont, Cat. No. 56001) in PBS-T. Sections were then blocked with 5% normal goat serum in PBS-T and incubated overnight with a mouse monoclonal anti-tyrosine hydroxylase (TH) antibody (Sigma, Cat. No. ZMS1033, 1:500) diluted in PBS-T containing normal goat serum. After washing, sections were incubated with a biotinylated secondary antibody (Vector, Cat. No. BA-9200, 1:500) followed by HRP-conjugated streptavidin (Invitrogen, Cat. No. 43-4323, 1:5000) at room temperature. Immunoreactivity was visualized with diaminobenzidine (DAB) and 0.3% H_2_O_2_ for 30 min. Sections were then mounted on gelatin-coated slides, air-dried, dehydrated through graded ethanol, cleared in xylene, and coverslipped with Entellan mounting medium. Control sections were processed to verify antibody performance. Images were acquired with a Leica MZ12 stereomicroscope equipped with 1X or 2X objectives.

Except for the primary antibody incubation, immunofluorescence procedures followed the initial tissue processing steps described above for immunohistochemistry. L5 spinal cord sections were incubated overnight at 4 °C in PBS containing a cocktail of the primary antibodies: rabbit monoclonal anti-NF-κB/p-p65 (1:500, Cat. No. 3033, Cell Signaling Technology), and goat polyclonal anti-Iba1 (1:5000, Cat. No. ab107159, Abcam). After rinsing in 0.1 M PBS, sections were incubated for 24 h at 4 °C in the dark with a secondary antibody cocktail consisting of donkey anti-rabbit Alexa Fluor 488 (1:300, Cat. No. A21206, Thermo Fisher Scientific), donkey anti-goat Alexa Fluor 555 (1:300, Cat. No. A21432, Thermo Fisher Scientific) combined with a directly conjugated mouse monoclonal anti-glial fibrillary acidic protein GFAP-Alexa Flour 647 (1:1000, Cat. No. 3657 S, Cell Signaling Technology). To verify antibody specificity, negative control sections were processed in parallel by omitting the primary antibodies.

Epifluorescence images were acquired using a motorized inverted Olympus IX81 fluorescence microscope (Olympus Latin America, Inc., FL, USA) equipped with a fiber-coupled xenon lamp (Cell-R DSU module, Olympus). Optical sections were captured at a resolution of 1324 × 1024 pixels (corresponding to a field of view of 563 × 407 μm) and averaged over time to optimize the signal-to-noise ratio. High-magnification images (10x) of the labeled cells were acquired with an ORCA-R2 digital CCD camera (Hamamatsu Photonics K.K., Japan) managed by xCellence-RT imaging software (version 1.2; Olympus Soft Imaging Solutions GmbH, Germany).

For quantitative assessment, triplicate sections were analyzed per rat to evaluate GFAP^+^, Iba1^+^, and NF-κB/p-p65^+^ cellular profiles. The distribution and labeling of NF-κB/p-p65, GFAP, and Iba1 within L5 spinal cord cells were evaluated using a semi-automatic MD3 Image Processing System (Minnesota Datametrics) with Data Image Tracer software (version 1.2), alongside ImageJ/Fiji software (NIH, Bethesda, MD, USA). Within each image, the boundaries of the first five laminae of the L5 spinal cord dorsal horn were delineated. Images were processed using the MultiPoint tool. The pixel area for each lamina was determined and converted to square millimeters (mm2) based on the total calibrated area. Positive cells were pooled by anatomical region, grouping laminae I-II and laminae III-IV for each marker per section. Finally, cellular density was calculated by dividing the total number of positive cells by the respective area of the analyzed laminae group, with data expressed as the number of positive cells per square millimeter (cell/mm^2^).

### ELISA Multiplex

To determine the protein expression of IL-1β and TNF-α, L4-L6 spinal cord tissues from 6-OHDA-lesioned rats were processed using a commercial multiplex magnetic bead-based immunoassay kit (MILLIPLEX MAP Rat Cytokine/Chemokine Magnetic Bead Panel, Cat. No. RECYTMAG-65 K; MilliporeSigma, Burlington, MA, USA). All assay procedures, including sample incubation, washing steps, and standard curve preparations, were performed in strict accordance with the manufacturer’s instructions. The plates were analyzed using the Luminex system (Luminex 200, equipped with Luminex xPONENT software). To account for variations in sample size and tissue extraction efficiency, total protein concentration for each spinal cord lysate was determined using a commercial Bradford method protein assay kit. Final cytokine concentrations were normalized to the total protein content and expressed as picograms of cytokine per milligram of total protein (pg/mg protein).

### Experimental Design

This study used a total of 106 adult male rats. Baseline assessments of tactile allodynia and mechanical hyperalgesia were performed before rats were randomly assigned to the following initial groups: naive, sham, or 6-OHDA, using a computer-generated randomization sequence to ensure an unbiased distribution. Fourteen days after surgery, successful induction of the PD model by unilateral 6-OHDA injection into the SNpc was confirmed with the cylinder test, followed by the apomorphine-induced turning test on day 15. An open-field test was performed at 20–25 days post-surgery to assess whether rotigotine administration caused motor impairment or hyperlocomotion, thereby rigorously excluding any potential pharmacological confounding effects of the dopamine agonist on subsequent pain-related behavioral outcomes (Fig. 1A).

To evaluate the antiallodynic and antihyperalgesic effects of rotigotine, an acute treatment protocol was initiated 20 days after surgery (Fig. 1A). Animals received intranasal vehicle (0.9% saline) or rotigotine (0.1 [*n* = 6], 0.5 [*n* = 8], 0.75, or 1 mg/kg; *n* = 6 per group for the remaining doses). An additional cohort of vehicle-treated naïve and sham-operated rats (*n* = 6 per group) was maintained to establish baseline thresholds. The intranasal dose of rotigotine was selected based on preliminary experiments in our laboratory, which established this range as the optimal therapeutic window to evaluate antiallodynic and antihyperalgesic effects without inducing baseline motor deficits. The intranasal administration was performed using a micropipette to deliver a total volume of 50 µL per animal (25 µL per nostril). Rats were gently restrained in an anti-Trendelenburg position, and the solutions were delivered alternately between the nares in small droplets, allowing each drop to be completely inhaled through natural respiration. To ensure consistent and uniform drug delivery, rats were maintained in this position for an additional 1 min to prevent fluid loss or swallowing. An independent researcher encoded treatment groups so the investigator performing the behavioral assessments remained blinded to the drug and dose administered. Tactile allodynia and mechanical hyperalgesia were assessed at 0, 2, 4, 6, and 8 h after administration. For subacute treatment (days 21–25 post-surgery), the same groups received daily intranasal administration of rotigotine or vehicle for five consecutive days, and pain-related behaviors were assessed before each daily dose. On day 26, animals were euthanized, and tissues were fixed in 4% paraformaldehyde (Fig. 1B) for subsequent analyses.

To determine the dopaminergic mechanisms D1/D2/D3 receptors underlying the antiallodynic and antihyperalgesic effects of rotigotine, 6-OHDA-lesioned rats received intranasal pretreatment with the D1 receptor antagonist SCH-23,390 (3 and 30 nmol, *n* = 6 per group; Ki = 0.3) [21], the D2 receptor antagonist L-741,626 (1.46 and 14.6 nmol, *n* = 6 per group; Ki = 2.4) [22], or the D3 receptor antagonist nafadotride (0.3 and 3 nmol, *n* = 6 per group; Ki = 0.52) [23] once daily for five consecutive days, 10 min before each daily administration of an effective dose of rotigotine (0.5 mg/kg, i.n.). The highest dose of each antagonist was also administered alone to evaluate possible intrinsic effects on nociceptive thresholds (*n* = 6 per group). As with the prior protocol, allocations and treatments for the antagonist study were randomized and fully blinded to the observer. Tactile allodynia and mechanical hyperalgesia were assessed daily before each administration. For the antagonist subacute protocol, the overall antiallodynic and antihyperalgesic effects were summarized as percentage of maximum possible effect (%MPE), calculated from normalized area-under-the-curve (AUC) values obtained across the 5-day treatment period. On day 26, animals were euthanized and transcardially perfused with 4% paraformaldehyde. Brain and spinal cord tissues were collected and stored at 4 °C–− 70 °C for subsequent immunohistochemical, immunofluorescence, and ELISA analyses, respectively.

The allocation and detailed distribution of the 106 rats across all experimental phases—including the 20 rats utilized for the initial dose-response protocol, the 54 rats assigned to the 9 distinct dopaminergic antagonist groups (*n* = 6 per group), and the baseline control/vehicle cohorts—are graphically represented in the experimental flowchart (Fig. 1B).

### Sample Size Justification

The sample size of *n* = 6 to *n* = 8 animals per group was determined a priori using a statistical power analysis performed via G*Power software (v3.1). For the primary behavioral outcomes (Cylinder test and Turning behavior) and neurochemical assays, group size was calculated using an F-test (ANOVA). With a significance level (α) of 0.05 and a target statistical power (1-β) of 80%, the analysis indicated that a minimum sample size of *n* = 6 per group is statistically sufficient to detect large and biologically meaningful effect sizes (Cohen´s f ≈ 0.04) typically observed in 6-OHDA models of neurodegeneration. Furthermore, the total initial enrollment (*N* = 106) incorporated a predicted 10–15% attrition and surgical mortality buffer, ensuring that the final cohort size per group did not drop below the minimum required threshold for reliable parametric inferential statistics.

For immunofluorescence, astrocytes were identified by glial fibrillary acidic protein (GFAP; *n* = 4) and microglia were identified by ionized calcium-binding adaptor molecule 1 (Iba1; *n* = 4). In addition, NF-κB/p-p65 (Ser536) expression was evaluated (*n* = 4) using an antibody that specifically recognizes the phosphorylated Ser536 residue without cross-reactivity with the p50 subunit or related proteins. Moreover, spatial relationships and regional association between NF-κB/p-p65 and glial markers (GFAP and Iba1) were evaluated within the L5 spinal cord dorsal horn. TH immunohistochemistry was also performed to quantify dopaminergic depletion induced by the 6-OHDA lesion (*n* = 4 per group) (Fig. 1). Furthermore, TNF-α and IL-1β levels were quantified by ELISA in the L4–L6 ipsilateral portion of the spinal cord (*n* = 4). To maintain objectivity, microscopic images for immunohistochemistry and immunofluorescence were captured by an independent investigator who was blinded and separated from the researcher performing the subsequent quantification. Furthermore. All tissue analyses (including image quantification and ELISA optical densities) were conducted by a blinded investigator unaware of the experimental groups; decoding was performed only during the final data analysis stage. Because rotigotine at 0.5 mg/kg produced robust antiallodynic and antihyperalgesic effects without inducing baseline motor deficit, this dose was selected to evaluate whether rotigotine modulates NF-κB/p-p65 activation in astrocytes and microglia, as well as the release of proinflammatory cytokines such as TNF-α and IL-1β.

### Molecular Docking Studies

To determine the potential binding mode of rotigotine, we used the NF-κB structural model obtained from the Protein Data Bank (PDB ID: 1NFI), representing the IκBα-bound conformation of NF-κB (chains A and C, p65 subunit; chains B and D, p50 subunit; chains E and F, IκBα) [24]. This structure was selected based on its resolution (2.7 Å) and prior use in small-molecule docking studies targeting NF-κB [25, 26]. Water molecules were removed using PyMOL version 2.4.0. The ligands caffeic acid phenethyl ester (CAPE; PubChem CID: 5281787) and rotigotine (PubChem CID: 59227) were downloaded from the PubChem database. Prior to docking, the protonation state of all compounds was assigned at a physiological pH of 7.4, and their 3D geometries were optimized using YASARA Structure (version 24.10.5) [27, 28].

Docking calculations were performed using AutoDock and AutoDock Vina, both implemented in the YASARA Structure suite (version 24.10.5) [27, 28]. Initially, blind docking was performed by defining a simulation cell (grid box) that covered the entire NF-κB/IκBα complex with an additional 5 Å padding. The prepared protein and simulation cell were stored as a .sce file. Screening was then conducted with both docking programs using the protein (.sce file) and ligand (*.sdf file) inputs via the dock_runscreening macro, adapted to perform 100 runs of the Lamarckian Genetic Algorithm. Analysis of the resulting binding poses revealed that both ligands converged predominantly to a common binding site in chain C, within the C-terminal region of the p65 Rel homology domain, proximal to the p65 dimerization and IκBα-binding interface. Based on this finding, a second round of site-specific molecular docking was conducted, defining a focused simulation cell with a 2 Å extension around the previously identified binding pose. For AutoDock, 100 Lamarckian Genetic Algorithm runs were executed via the dock_run macro, while AutoDock Vina was configured to perform 500 runs. The binding pose exhibiting the largest cluster size and the most favorable binding energy was selected for subsequent analysis.

CAPE, a reported NF-κB inhibitor, was docked under the same conditions as rotigotine for computational benchmarking purposes. As AutoDock and AutoDock Vina model non-covalent interactions, this protocol does not simulate CAPE’s reported covalent mechanism of action (cysteine alkylation within the p50 subunit); accordingly, CAPE was used solely as a computational reference compound, evaluated under identical docking conditions to rotigotine, rather than as a mechanistic positive control.

### Quantitative Regional Correlation Analysis

To evaluate the spatial relationship and regional association between NF-κB/p-p65 and glial markers (GFAP and Iba1) within the L5 spinal cord dorsal horn, quantitative correlation analysis was performed using ImageJ/Fiji software (NIH, Bethesda, MD, USA) via the Coloc 2 plugin in 4 rats per experimental group, evaluating 3 spinal cord sections per rat. Standardized regions of interest (ROIs) encompassing laminae I-IV were defined within captured fields with a fixed-dimension area of 563 × 407 μm. After background subtraction, the spatial overlap was expressed as Pearson´s correlation coefficient (r), which serves as a dimensionless metric ranging from − 1 (perfect negative correlation) to + 1 (perfect positive correlation), calculated to determine the linear relationship of signal intensity distributions between channels without prior threshold segmentation. Given the spatial resolution limits of the Olympus IX81 fluorescence microscope (Olympus Latin America, Inc., Florida, USA) at 10x magnification, these resulting values were interpreted strictly as macro-level regional tissue co-distributions and signal associations within the dorsal horn microenvironment. This analysis deliberately avoids verifying definitive macromolecular intracellular colocalization, which would require optical sectioning techniques.

### Statistical Analysis

Data are presented as mean ± standard error of the mean (SEM). Statistical analyses and graphs were generated using GraphPad Prism version 8.0.1 (GraphPad Software, San Diego, CA, USA). To control the family-wise error rate across multiple experimental conditions, appropriate post hoc tests were strictly applied to all multi-group analyses. Single-factor group comparisons, including immunofluorescence measurements, regional Pearson correlation coefficients, cylinder-test measurements, apomorphine-induced turning behavior, and TH-positive neuronal counts, were analyzed by one-way ANOVA followed by Tukey’s post hoc test for multiple pairwise comparisons. Longitudinal or multifactorial data, including the 50% paw withdrawal threshold, the number of hind paw withdrawal responses, and open-field performance across treatment days, were analyzed by repeated-measures two-way ANOVA followed by Bonferroni’s post hoc test to control for multiple comparisons across groups at individual time points. For the subacute antagonist protocol, behavioral responses across the 5-day treatment period were normalized and integrated as area-under-the-curve values and expressed as a percentage of the maximum possible effect for allodynia and hyperalgesia. These summary values were analyzed by one-way ANOVA followed by Tukey’s post hoc test. For all analyses, reported significance levels correspond to final adjusted P values, and a P value < 0.05 was considered statistically significant.

## Results

### Validation of the Unilateral 6-OHDA-induced Parkinsonian Model

**Fig. 2:**
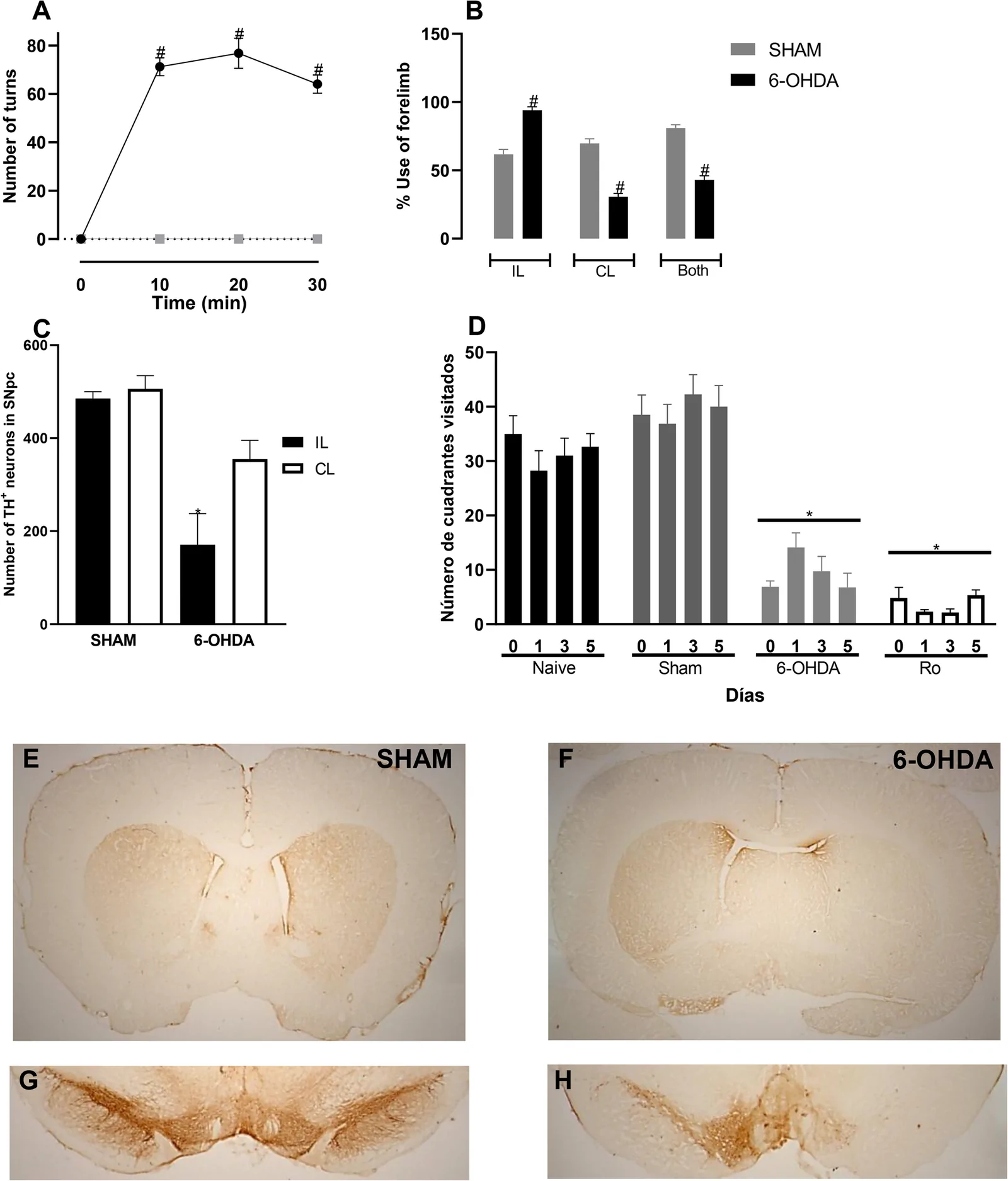
Validation of the unilateral 6-OHDA-induced Parkinsonian model in rats.** A** Number of contralateral turns during 30 min after apomorphine administration (0.1 mg/kg, s.c.) 15 days after surgery. **B** Percentage use of the ipsilateral (IL), contralateral (CL), and both forelimbs in the cylinder test 14 days after unilateral 6-OHDA lesion of the SNpc. **C** Number of tyrosine hydroxylase-positive (TH^+^) neurons in the SNpc. **D** Open-field performance evaluated on days 0, 1, 3, and 5 (starting 20 days post-surgery) in naive (N), sham (S), 6-OHDA, and Ro (rotigotine, 0.5 mg/kg) groups. Representative tyrosine hydroxylase immunostaining in the striatum (**E**,** F**) and SNpc (**G**,** H**) of sham and 6-OHDA-lesioned rats. Data are presented as mean ± SEM (*n* = 6 per group). ^#^*P* < 0.05 sham vs. 6-OHDA, as determined by two-way ANOVA followed by Tukey’s post hoc test. ^&^*P* < 0.05 IL sham vs. 6-OHDA, as determined by one-way ANOVA followed by Tukey´s post hoc test. *** *P* < 0.001 sham vs. 6-OHDA and Ro as determined by two-way ANOVA followed by Bonferroni´s post hoc test

Unilateral injection of 2 µL of 6-OHDA at 8 µg/µL (total dose, 16 µg) into the SNpc successfully induced a Parkinsonian phenotype. In the apomorphine-induced rotation test, lesioned rats exhibited a marked increase in contralateral turning behavior (202 ± 22 turns/30 min), whereas the sham group showed no rotations (two-way repeated-measures ANOVA revealed significant effects of group *F*_(1, 18)_ = 337.8, *p* < 0.0001, time *F*_(3, 54)_ = 115.8, *p* < 0.0001, and the group × time interaction *F*_(3, 54)_ = 115.8, *p* < 0.0001 followed by Bonferroni´s post hoc test, Fig. 2A). Motor asymmetry was further confirmed in the cylinder test, in which 6-OHDA-lesioned rats showed a significant reduction in the simultaneous use of both forelimbs (43.13%) compared with the sham group (82.25%) and the use of CL forelimbs (30%) compared with the sham group (two-way ANOVA, *F*_(3,12)_ = 387.8, *p* < 0.0001, followed by Bonferroni’s post hoc test; Fig. 2B). In addition, locomotor activity in the open-field test was markedly impaired in the 6-OHDA group, which showed fewer crossings (10 crossings) than the sham group (35 crossings) (one-way ANOVA, *F*_(15,104)_ = 27.20, *p* < 0.0001, followed by Tukey’s post hoc test; Fig. 2D). 

**Fig. 3:**
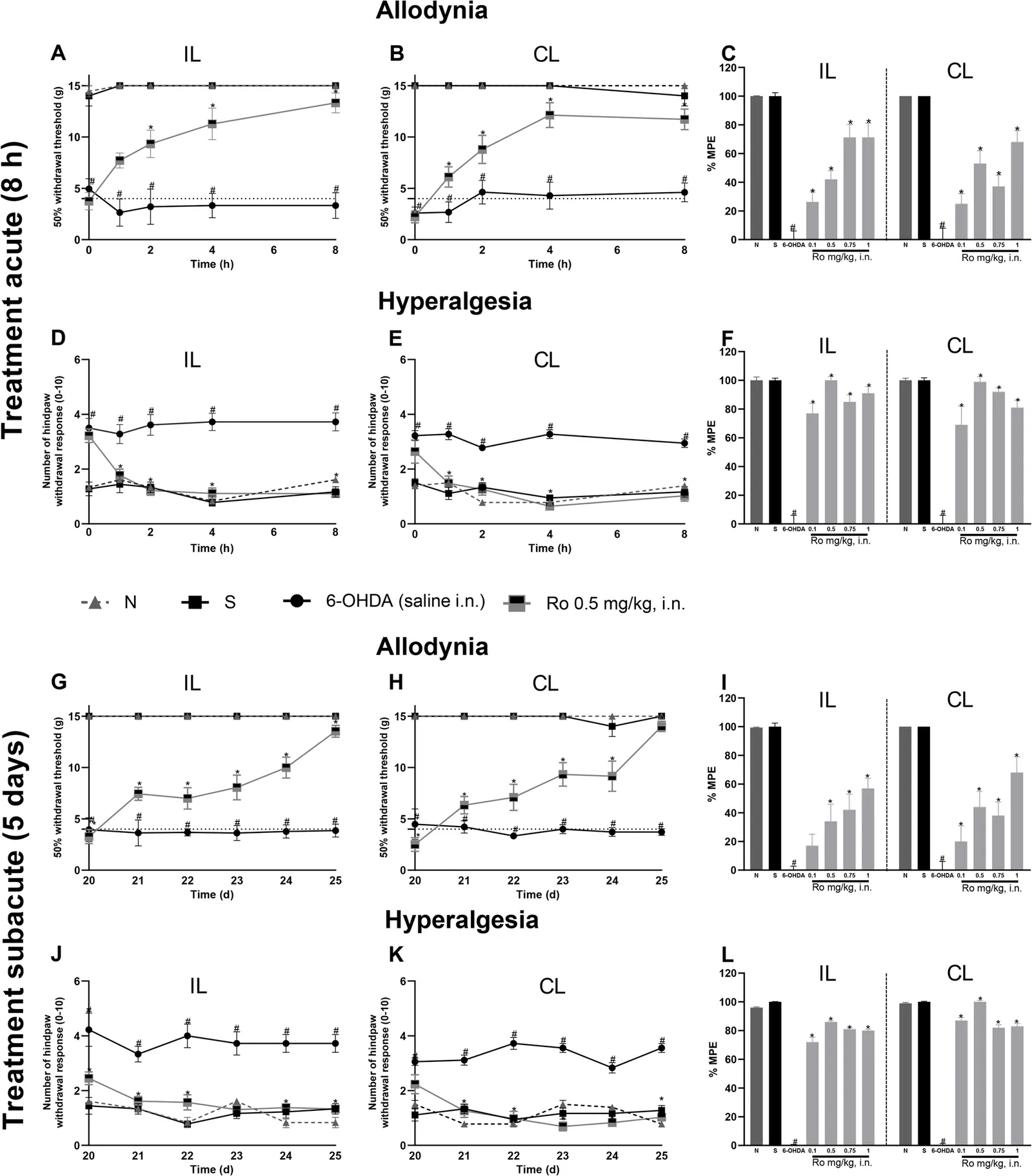
Antiallodynic and antihyperalgesic effects induced by acute (8 h) and subacute (5 days) intranasal administration of rotigotine. **A–F **Acute treatment: Twenty days after unilateral lesion with 2 µL of 6-OHDA at 8 µg/µL (total dose, 16 µg), tactile allodynia (**A **and** B**) and mechanical hyperalgesia (**D **and** E**) were evaluated in the ipsilateral (IL) and contralateral (CL) hind paws at 0, 2, 4, 6, and 8 h after administration. The percentage of maximum possible effect (%MPE) was determined for allodynia (**C**) and hyperalgesia (**F**). **G–L **Subacute treatment (5 days): tactile allodynia (**G **and** H**) and mechanical hyperalgesia (**J **and** K**) were assessed in the IL and CL paws every 24 h before daily rotigotine or vehicle administration, where the x-axis indicates days 20 to 25 post-lesion (with day 20 serving as the baseline before the 5-day subacute protocol. %MPE was calculated for allodynia (**I**) and hyperalgesia (**L**). Data are presented as mean ± SEM (*n* = 6 per group). ^#^*P* < 0.05 sham vs. 6-OHDA; *P < 0.05 6-OHDA vs. treated groups, as determined by one-way or repeated-measures two-way ANOVA followed by Bonferroni’s post hoc test. N: naive; S: sham

Consistent with these behavioral deficits, rats with unilateral 6-OHDA lesions exhibited a marked loss of TH immunoreactivity. Dopaminergic degeneration was quantified by counting TH-positive (TH^+^) neurons in the SNpc. The 6-OHDA group showed a substantial reduction in the number of TH^+^ neurons (170) compared with the sham group (500) (one-way ANOVA, *F*_(3,12)_ = 53.69, *p* < 0.0001, followed by Tukey’s post hoc test; Fig. 3C). Representative images further illustrate the pronounced depletion of dopaminergic neurons in both the striatum and the SNpc of 6-OHDA-lesioned rats (Fig. 3F and H) compared with the preserved TH staining observed in the sham group (Fig. 3E and G). Overall, these data indicate an approximately 80% loss of dopaminergic neurons in 6-OHDA-lesioned rats relative to sham controls.

### Antiallodynic and Antihyperalgesic Effects of Acute and Subacute (5-day) Intranasal Rotigotine Treatment in 6-OHDA-lesioned Rats

Unilateral 6-OHDA lesion of the SNpc significantly reduced the 50% paw withdrawal threshold in both the IL and CL hind paws compared with the sham group (Fig. 3A–B). Acute intranasal rotigotine (0.1–1 mg/kg), but not vehicle, dose-dependently increased the 50% paw withdrawal threshold in both paws from 2 to 8 h post-administration, peaking at 4 h. Two-way ANOVA for the IL paw showed significant main effects of [group *F*_(6, 35)_ = 78.38, *p* < 0.0001, time *F*_(4, 140)_ = 21.67, *p* < 0.0001, and group x time interaction *F*_(24, 140)_ = 3.86, *p* < 0.0001], similarly, the CL paw analysis revealed significant effects for group [*F*_(6, 35)_ = 87.57, *p* < 0.0001, time *F*_(4, 140)_ = 33.19, *p* < 0.0001, and interaction *F*_(24, 140)_ = 4.50, *p* < 0.0001]. At 1 mg/kg, rotigotine produced a %MPE of 72% in the IL paw [one-way ANOVA *F*_(6, 35)_ = 2939, *p* < 0.0001] and 68% in the CL paw [one-way ANOVA *F*_(6, 35)_ = 1203, *p* < 0.0001] (Fig. 3C).

Regarding mechanical hyperalgesia, 6-OHDA-lesioned rats exhibited more withdrawal responses to a 250 mN von Frey filament than sham animals in both paws. Acute rotigotine significantly reduced these responses, showing a similar profile with a peak at 4 h and effects lasting at least 8 h. Two-way ANOVA confirmed significant effects in the IL paw [group *F*_(6, 35)_ = 40.57, *p* < 0.0001, time *F*_(4, 140)_ = 26.15, *p* < 0.0001 and interaction *F*_(24, 140)_ = 4.66, *p* < 0.0001] and the CL paw, [group *F*_(6, 35)_ = 28.40, *p* < 0.0001, time *F*_(4, 140)_ = 25.10, *p* < 0.0001 and interaction *F*_(24, 140)_ = 2.70, *p* < 0.0001]. At 1 mg/kg, the %MPE was 85% in the IL paw [one-way ANOVA *F*_(6, 35)_ = 1038, *p* < 0.0001] and 80% in the CL paw [*F*_(6, 35)_ = 2691, *p* < 0.0001] (Fig. 3F).

Subacute intranasal rotigotine (0.1–1 mg/kg, 5 days) sustained the antiallodynic effect, reaching maximal efficacy on day 4. Two-way ANOVA indicated significant effects for the IL paw, [group *F*_(6, 35)_ = 82.47, *p* < 0.0001, time *F*_(6, 210)_ = 41.17, *p* < 0.0001 and interaction *F*_(36, 210)_ = 5.27, *p* < 0.0001] and the CL paw, [group *F*_(6, 35)_ = 78.52, *p* < 0.0001, time *F*_(6, 210)_ = 38.91, *p* < 0.0001, and interaction *F*_(36, 210)_ = 5.70, *p* < 0.0001]. This yielded a day 4%MPE of 72% (IL) [one-way ANOVA, F _(6, 35) =_ 2020, *p* < 0.0001] and 68% (CL) [one-way ANOVA, *F*_(6, 35)_ = 2709, *p* < 0.0001] (Fig. 3I). Likewise, subacute treatment exerted significant antihyperalgesic effects from day 1 through day 5 (maximal on day 4). Two-way ANOVA revealed significant effects for the IL paw [group *F*_(6, 35)_ = 51.81, *p* < 0.0001, time *F*_(6, 210)_ = 38.81, *p* < 0.0001, and interaction *F*_(36, 210)_ = 5.07, *p* < 0.0001] and the CL paw [group *F*_(6, 35)_ = 54.70, *p* < 0.0001, time *F*_(6, 210)_ = 29.91, *p* < 0.0001, and interaction *F*_(36, 210)_ = 6.39, *p* < 0.0001]. At 1 mg/kg, the %MPE was 57% (IL) [one-way ANOVA, *F*_(6, 35)_ = 1470, *p* < 0.0001] and 68% (CL) [one-way ANOVA, *F*_(6, 35)_ = 1945, *p* < 0.0001] (Fig. 3L). Together, these findings support the sustained efficacy of intranasal rotigotine in reducing mechanical hypersensitivity in 6-OHDA-lesioned rats.

### Involvement of D2 and D3, but not D1, Dopaminergic Receptors in Rotigotine-induced Antiallodynic and Antihyperalgesic Effects

**Fig. 4:**
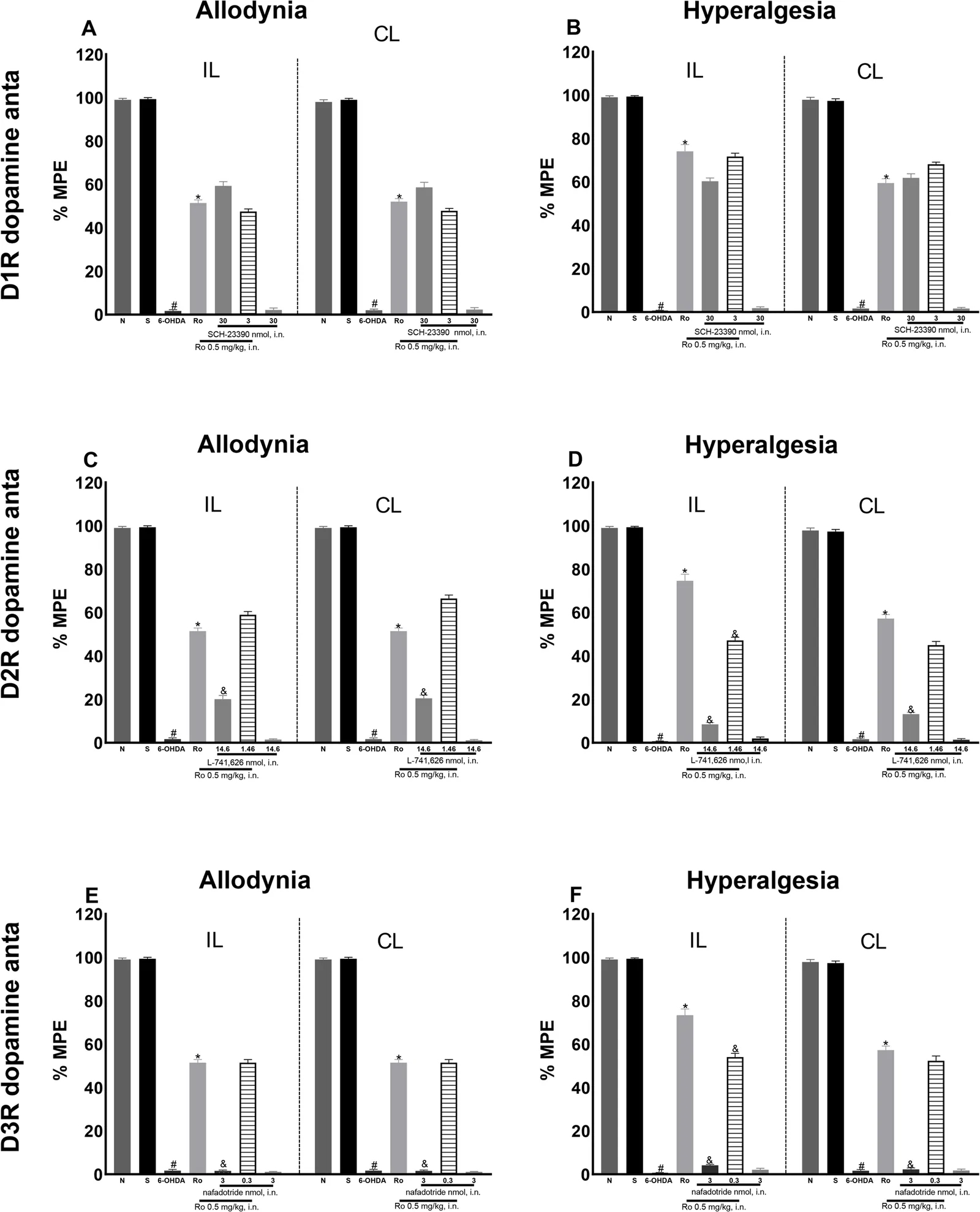
Dopaminergic receptors mediate the antiallodynic and antihyperalgesic effects of rotigotine (0.5 mg/kg, i.n.). Twenty days after unilateral 6-OHDA lesion, tactile allodynia and mechanical hyperalgesia were evaluated to determine the involvement of specific dopamine receptors. In the subacute protocol, selective antagonists were administered intranasally once daily for 5 days, 10 min before each effective dose of rotigotine (0.5 mg/kg, i.n.): D1 receptor antagonist SCH-23390 (3 and 30 nmol, i.n.; **A**, **B**), D2 receptor antagonist L-741, 626 (1.46 and 14.6 nmol, i.n.; **C**, **D**), or D3 receptor antagonist nafadotride (0.3 and 3 nmol, i.n.; **E**, **F**). Pain-related behaviors were assessed daily before each administration. Antiallodynic and antihyperalgesic effects are expressed as a percentage of the maximum possible effect (%MPE), calculated from normalized area under the curve (AUC) values across the 5-day treatment period. Data is presented as mean ± SEM (n = 6 per group). #P < 0.05 sham vs. 6-OHDA; *P < 0.05 6-OHDA vs. treated groups, as determined by one-way ANOVA followed by Tukey’s post hoc test. IL: ipsilateral; CL: contralateral; N: naive; S: sham

To elucidate the mechanisms underlying rotigotine’s antinociceptive action, we evaluated selective dopamine receptor antagonists as part of a subacute treatment protocol. Daily intranasal treatment with the selective D1 receptor antagonist SCH-23,390 (3 and 30 nmol) for five consecutive days, administered 10 min before each dose of rotigotine (0.5 mg/kg, i.n.), failed to reverse the sustained antiallodynic effects in both the IL paw [one-way ANOVA, *F*_(6,35)_ = 978.2, *p* < 0.0001] and CL paw [one-way ANOVA, *F*_(6,35)_ = 869.8, *p* < 0.0001], followed by Tukey’s post-hoc test. Similarly, D1 receptor blockade did not alter rotigotine-induced antihyperalgesic effects in the IL paw [one-way ANOVA, *F* (_6,35_) = 1640, *p* < 0.0001] or CL paw [one-way ANOVA, *F*_(6,35)_ = 634.6, *p* < 0.0001]. Furthermore, SCH-23,390 alone (30 nmol) had no significant effect on nociceptive thresholds (Fig. 4A–B). In contrast, repeated treatment with the selective D2 receptor antagonist L-741,626 at 14.6 nmol (but not 1.46 nmol) partially reversed the antiallodynic effects in the IL paw [one-way ANOVA, *F*_*(*6,35)_ = 1171, *p* < 0.0001] and CL paw [one-way ANOVA, *F*_(6,35)_ = 1162, *p* < 0.0001], as well as the antihyperalgesic responses in the IL paw [one-way ANOVA, *F*_(6,35)_ = 3004, *p* < 0.0001] and CL paw [one-way ANOVA, *F*_(6,35)_ = 952.7, *p* < 0.0001; Fig. 4C–D]. Likewise, repeated treatment with the selective D3 receptor antagonist nafadotride (3 nmol, but not 0.3 nmol) significantly reversed the antiallodynic effects in the IL paw [*F*_(6,35)_ = 1403, *p* < 0.0001] and CL paw [*F*_(6,35)_ = 1190, *p* < 0.0001], and the antihyperalgesic effects in the IL paw [*F*_(6,35)_ = 2980, *p* < 0.0001] and CL paw [*F*_(6,35)_ = 852.9, *p* < 0.0001; Fig. 4E–F]. Nafadotride alone (3 nmol) also showed no effect on baseline nociceptive responses. Collectively, these findings indicate that the sustained analgesic effects of intranasal rotigotine are mediated primarily through D2 and D3 receptor activation, whereas D1 receptors do not appear to play a major role.

### In Silico Analysis of Rotigotine Interaction with NF-κB

We performed molecular docking simulations to explore potential interactions between rotigotine and NF-κB (PDB ID: 1NFI). CAPE, a reported NF-κB inhibitor, was also docked under the same conditions as a computational reference.

**Fig. 5:**
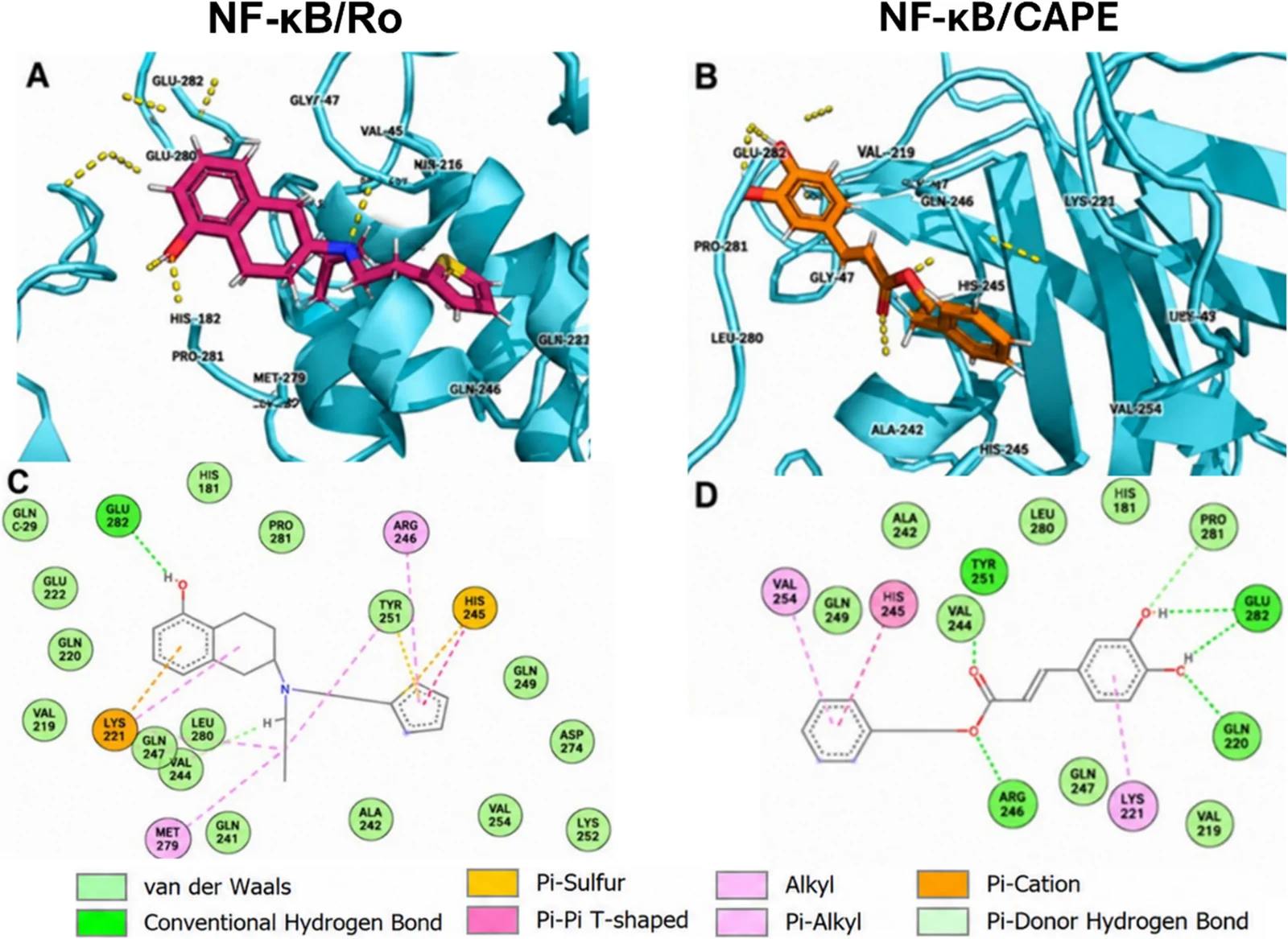
Molecular docking: binding interactions of NF-κB with rotigotine and CAPE. Molecular docking: binding mode of rotigotine (**A**) and CAPE (**B**) docked into the binding site of NF-κB (cyan). 2D diagrams of rotigotine (**C**) and CAPE (**D**) docked into NF-κB, showing interactions with residues at the binding site

Docking analysis predicted a theoretical binding pose in which rotigotine may interact with NF-κB through hydrophobic contacts involving Leu^280^, Gly^247^, and Pro^241^ (Fig. 5A and C), located within the C-terminal region of the p65 Rel homology domain. CAPE showed interactions with Leu^280^, Gly^249^, His^181^, and Lys^252^ within the same region (Fig. 5B and D); notably, this docked pose does not correspond to CAPE’s reported experimental site of covalent action within the p50 subunit. Rotigotine demonstrated favorable docking scores of − 10.13 kcal/mol in AutoDock 4.2 and − 7.22 kcal/mol in AutoDock Vina, whereas CAPE showed docking scores of − 9.81 kcal/mol and − 8.28 kcal/mol, respectively. The main interaction sites for rotigotine were in chains A and C, corresponding to the p65 subunit of NF-κB. These in silico findings suggest a candidate binding mode of rotigotine at NF-κB. However, because docking predictions alone cannot establish direct binding or receptor-ligand interactions, this hypothesis requires experimental validation using biophysical binding assays and functional studies.

### Unilateral 6-OHDA Lesion of the SNpc Induces Glial Activation and NF-κB/p-p65 Upregulation in the L5 Spinal Cord Dorsal Horn

**Fig. 6:**
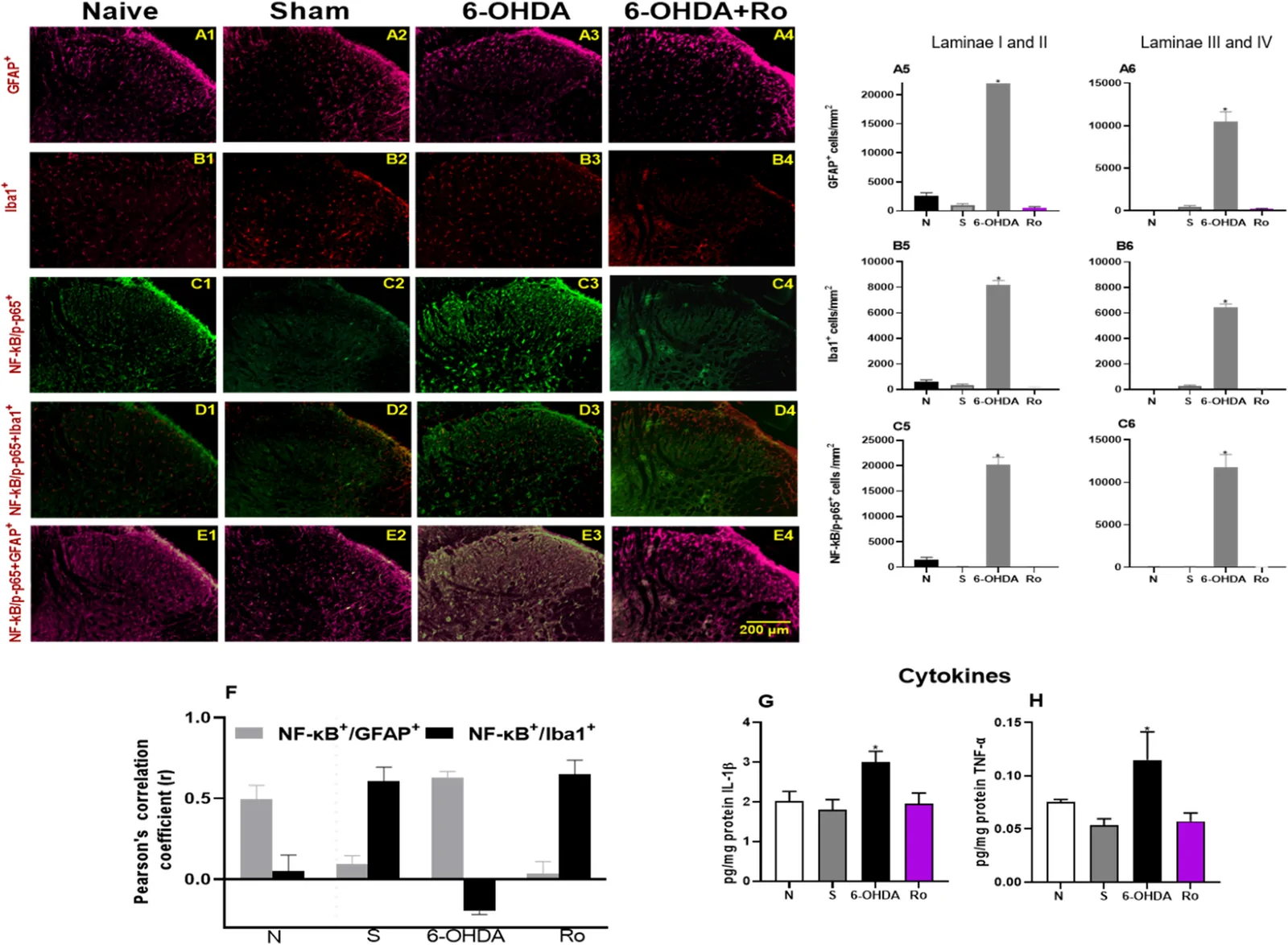
Immunostaining, quantitative regional correlation, and cytokine expression in the L5 spinal cord dorsal horn of rats with unilateral 6-OHDA lesions. A1–A4 Representative images of glial fibrillary acidic protein (GFAP)-immunoreactive astrocytes. B1–B4 Representative images of ionized calcium-binding adaptor molecule 1 (Iba1)-positive microglia. C1–C4 Representative images of phosphorylated NF-κB/p-p65 expression. A5, A6 Quantification of GFAP+ cells per mm² in laminae I–II and III–IV, respectively. B5, B6 Quantification of Iba1+ cells per mm² in laminae I–II and III–IV, respectively. C5, C6 Quantification of NF-κB/p-p65+ cells per mm² in laminae I–II and III–IV, respectively. 6D1-D4 Representative images of merge between NF-κB/p-p65+ and Iba1+ cells. 6E1-E4 Representative images of merge between NF-κB/p-p65+ and GFAP+ cells. Images were acquired using a 10X objective. Spinal cord cytokine expression in 6-OHDA-lesioned animals: **F** IL-1β and (**G**) TNF-α levels were quantified in the L4–L5 lumbar spinal cord section and expressed as pg/mg of total protein. **H** Quantitative regional correlation analysis showing mean Pearson´s correlation coefficient (r) for NF-κB/p-p65 signal distribution within GFAP+ astrocytic domains and Iba1+ microglial domains across experimental groups. Data are presented as mean ± SEM (n = 4 per group). #P < 0.05 sham vs. 6-OHDA; *P < 0.05 6-OHDA vs. treated groups, as determined by one-way ANOVA followed by Tukey’s post hoc test

Unilateral 6-OHDA lesion of the SNpc significantly increased the number of GFAP^+^ astrocytes (Fig. 6A5–A6), Iba1^+^ microglia (Fig. 6B5–B6), and showed a marked increase in the overall NF-κB/p-p65^+^ immunoreactivity intensity (Fig. 6C5–C6) in the ipsilateral L5 spinal cord dorsal horn of 6-OHDA-lesioned rats compared with the sham group. Immunostaining revealed that these changes in glial profiles and p-p65 signal intensity were distributed across laminae I-IV, with greater intensity in the superficial laminae I-II. Merged immunofluorescence images suggested a spatial overlap of the regional NF-κB/p-p65 signal within areas enriched with Iba1^+^ microglia (Fig. 6D1-D4) and GFAP^+^ astrocytes (Fig. 6E1-E4).

To quantitatively validate these spatial patterns, we performed a regional Pearson´s correlation coefficient (r) analysis. In the vehicle-treated 6-OHDA group, a strong positive correlation was confirmed between NF-κB/p-p65 and GFAP^+^ astrocytes (*r* = 0.6308), whereas a negative, uncoupled relationship was observed within Iba1^+^ microglia profiles (*r* = -0.1975). This marked cell-type asymmetry contrasted with the sham group, which displayed a weak correlation for astrocytes (*r* = 0.0950) and a strong positive correlation for microglia (*r* = 0.6083). While these regional variations do not imply direct intercellular or nuclear activation mechanisms due to the spatial resolution constraints of standard epifluorescence microscopy at 10x magnification and the absence of confocal Z-stack or orthogonal analysis, these quantitative findings show a robust, phenotype-specific tissue-level signal association. Consequently, these results show that the unilateral 6-OHDA lesion of the SNpc is associated with a prominent spinal glial response and targeted upregulation of regional NF-κB/p-p65 immunoreactivity, predominantly within astrocytic domains in areas critically involved in nociceptive processing.

### Subacute Rotigotine Treatment Reduces Glial Activation and NF-κB/p-p65 Expression and TNF-α and IL-1β Levels in the L5 Spinal Cord Dorsal Horn of 6-OHDA-lesioned Rats

Subacute treatment with rotigotine (0.5 mg/kg, i.n.) for five consecutive days significantly reduced the number of GFAP^+^ astrocytes (Fig. 6A5–A6) and Iba1^+^ microglia (Fig. 6B5–B6), while markedly decreasing the overall NF-κB/p-p65^+^ immunoreactivity intensity (Fig. 6C5–C6) in the L5 spinal cord dorsal horn of 6-OHDA-lesioned rats. Quantitative spatial correlation analysis revealed that rotigotine treatment completely abolished the signal association between NF-κB/p-p65 and GFAP^+^ astrocytes, reducing the Pearson´s coefficient to close to zero (*r* = 0.0358). Conversely, the treatment showed a robust positive correlation within microglial profiles (*r* = 0.6492), closely matching the microglial correlation observed in the sham condition. Although definitive intracellular colocalization or strict nuclear translocation of p-p65 within these specific glial phenotypes could not be histologically verified due to the aforementioned optical and resolution constraints, these findings qualitatively show that subacute rotigotine treatment effectively attenuates the spinal tissue-level NF-κB response while preserving or shifting the signal correlation towards microglial profiles in the dorsal horn of the L5 spinal cord.

Consistent with these findings, 6-OHDA-lesioned rats exhibited a significant increase in the spinal levels of TNF-α (one-way ANOVA, F _(3,12)_ = 3.9, *p* < 0.04, followed by Tukey´s post-hoc, Fig. 6G) and IL-1β (One-way ANOVA, F _(3,12)_ = 4.32, *p* < 0.03, followed by Tukey´s post-hoc test, Fig. 6F). Importantly, this upregulation was significantly reversed by the rotigotine treatment (0.5 mg/kg, i.n.) compared to the vehicle-treated 6-OHDA group.

## Discussion

### Unilateral 6-OHDA Lesion of the SNpc Induces Motor Deficits

The unilateral lesion with 6-OHDA (2 µL at 8 µg/µL; total dose, 16 µg) in the SNpc led to degeneration of dopaminergic neurons, as evidenced by a significant decrease of at least 80% in the number of TH^+^ neurons determined by immunostaining in the SNpc and a significant reduction in optical density in the striatum. Our data align with reports in the literature on 6-OHDA-lesioned animals in the SNpc, which demonstrate that 6-OHDA lesions (total dose, 16 µg) decrease the number of dopaminergic neurons in the SNpc and striatum [5, 19, 29–32]. Otherwise, a 6-OHDA lesion in the SNpc significantly increased the number of CL turns induced by apomorphine (0.1 mg/kg) [33, 34], reduced motor capacity, decreased the 71.2% of use of CL forelimb in the cylinder test, and decreased the number of crosses in the open field compared with the sham group, confirming the establishment of classical motor symptoms of PD. These data are consistent with a previous report in which a 6-OHDA-lesioned rat in the SNpc, with a total dose of 16 µg, showed a significant reduction in motor asymmetry, evidenced by decreased use of the CL limb and both legs, and decreased exploratory behavior [35, 36]. All data suggest that a 6-OHDA lesion in the SNpc causes loss of dopaminergic neurons and motor deficits, which characterize PD symptoms.

### Unilateral 6-OHDA Lesion in the SNpc Induced Long-Term Tactile Allodynia and Mechanical Hyperalgesia

Our study demonstrated that the unilateral 6-OHDA lesion (2 µL at 8 µg/µL; total dose, 16 µg) into the SNpc decreased the 50% paw withdrawal threshold and increased the number of hind paw withdrawal responses compared to the sham group, indicating tactile allodynia and mechanical hyperalgesia in both paws, respectively. These data agree with previous reports that 6-OHDA-lesioned animals exhibit hypersensitivity, including allodynia and hyperalgesia, in both paws [5, 19, 29, 31, 32]. Thus, our results confirm that unilateral 6-OHDA lesion in the SNpc induced long-term allodynia and hyperalgesia.

### Acute and Subacute Intranasal Rotigotine Treatment Alleviates Tactile Allodynia and Mechanical Hyperalgesia in 6-OHDA-lesioned Rats

In the present study, acute (8 h) and subacute (5 days) intranasal administration of rotigotine (0.5, 0.75, and 1 mg/kg) produced dose-dependent antiallodynic and antihyperalgesic effects, characterized by an increase in the 50% paw withdrawal threshold response and a decrease in the number of hind paw withdrawal responses, respectively. Maximum therapeutic effects were reached 4 h after acute intranasal administration and on day 4 with subacute treatment. Notably, our findings demonstrate for the first time that rotigotine reversed tactile allodynia and mechanical hyperalgesia in 6-OHDA-lesioned rats. Previous studies reported antinociceptive properties of rotigotine microspheres (RoMS; 5, 10, 20, and 40 mg/kg, i.m.) and increased the hindpaw withdrawal latency at days 3, 7, and 14 post-treatments in a carrageenan-induced inflammatory pain model [11]. Similarly, extended-release rotigotine microspheres (40 mg/kg i.m.) have been shown to decrease hindpaw withdrawal latency (HWL) in carrageenan-induced inflammatory pain [37]. Furthermore, clinical evidence shows that the rotigotine transdermal system significantly mitigates pain in patients with PD [10, 38, 39]. Interestingly, several authors have documented the antiallodynic and antihyperalgesic effects of dopamine agonists (levodopa, pramipexole, quinpirole, and rotigotine) in various animal pain models, including fibromyalgia induced by reserpine [40]; inflammatory pain [11, 41–43]; and PD pain induced by 6-OHDA [5, 19, 29]. However, the acute or subacute antinociceptive effects of rotigotine on neuropathic pain in the PD model remained unexplored. Consequently, our results provide the first direct evidence systematically characterizing the antiallodynic and antihyperalgesic effects of acute and subacute intranasal rotigotine administration in 6-OHDA-lesioned rats.

### D2 and D3 Receptors Mediate the Antiallodynic and Antihyperalgesic Effects of Rotigotine

In the present study, we examined the contribution of D1, D2, and D3 receptors to the antiallodynic and antihyperalgesic effects of rotigotine in 6-OHDA-lesioned rats. Our pharmacological results showed that pretreatment with the D1 receptor antagonist SCH-23,390 failed to reverse the sustained analgesic effects of rotigotine, whereas the D2 receptor antagonist L-741,626 partially attenuated these effects, and the D3 receptor antagonist nafadotride produced a more pronounced reversal. Together, these findings indicate that, at least at the studied doses of rotigotine (0.5 mg/kg, i.n.), activation of D2 and D3 receptors, but not D1 receptors, plays a central role in the analgesic actions of rotigotine in this model.

These findings are consistent with the known pharmacological profile of rotigotine, which exhibits higher affinity for D3 and D2 receptors than for D1 receptors [9]. This receptor profile may help explain why D3 receptor blockade produced the strongest reduction in rotigotine-induced antiallodynic and antihyperalgesic effects, whereas D2 receptor blockade produced only partial attenuation. Our interpretation is also in line with previous reports showing that the antinociceptive effects of rotigotine-loaded microspheres in inflammatory pain are mediated primarily through D2/D3 receptor activation [12] and with studies indicating that D2-like receptor signaling can modulate inflammatory and nociceptive processes [13, 44]. Although receptor affinity alone does not fully determine in vivo efficacy, the present results support the idea that D3 and D2 receptors are the principal dopaminergic mediators of rotigotine-induced analgesia in hemiparkinsonian rats. To rigorously support this interpretation and rule out off-target interactions, we selected antagonist doses within strict concentration thresholds based on their inhibition constants (Ki) and established functional profiles in neuroinflammation and pain models [21–23]. This pharmacological specificity is supported by our finding that the highly selective D1 receptor antagonist SCH-23,390 (Ki = 0.3 nM) failed to influence rotigotine-induced analgesia even at a high dose of 30 nmol, indicating that D1-like signaling is not required for this effect. In contrast, the D2 antagonist L-741,626 (Ki = 2.4 nM) and the preferential D3 antagonist nafadotride (Ki = 0.52 nM) exhibited clear dose-dependent reversals. At the lower doses tested (1.46 nmol for L-741,626 and 0.3 nmol for nafadotride), both compounds operated well within their selective ranges, targeting D2 and D3 receptors, respectively, with minimal risk of overlapping cross-reactivity. The progressive, enhanced reversal observed at higher doses (14.6 nmol and 3 nmol, respectively) reflects increased fractional receptor occupancy within nociceptive networks without inducing intrinsic pro-nociceptive effects, as the antagonist alone did not alter baseline thresholds. This clear dose-dependent profile reinforces the pharmacological specificity of our findings, demonstrating a cooperative or distinct contribution of both D2 and D3 receptor signaling pathways in modulating the neuroinflammatory and hyperalgesic cascades.

The spinal dorsal horn is a key site for integrating and modulating nociceptive information, and dopaminergic projections from the diencephalic A11 cell group contribute to descending inhibitory control of spinal pain processing [45]. Dopamine receptors are differentially distributed across dorsal horn laminae, suggesting that dopaminergic agonists may modulate nociceptive transmission through multiple receptor populations [46, 47]. Specifically, D2 receptors are predominantly localized in laminae II and III [46], whereas D3 receptors show their highest density in the superficial dorsal horn (laminae I and II) [48]. Given that these laminae are critical for gating and processing primary nociceptive inputs, the activation of local D2 and D3 receptor populations likely mediates the antiallodynic and antihyperalgesic effects of rotigotine observed in 6-OHDA-lesioned animals.

In this context, rotigotine-mediated activation of D2/D3 receptors may reduce spinal hyperexcitability not only through neuronal mechanisms but also through actions on glial cells, which express D2-like receptors and participate in neuroinflammatory signaling. Astrocytic and microglial D2 receptors generally suppress neuroinflammation through well-characterized intracellular pathways. Specifically, D2 receptor stimulation recruits β-arrestin 2, a G-protein-independent scaffold that forms a macromolecular complex with protein phosphatase 2 A (PP2A) and Akt. This interaction promotes the upregulation of PPP2R2C (a regulatory subunit of PP2A) and drives Akt dephosphorylation, effectively suppressing the downstream IKKα/IKKβ-IκB-NF-κB signaling cascade. By preventing IκBα phosphorylation, β-arrestin 2 sequesters the NF-κB subunit in the cytoplasm, blocking its nuclear translocation and subsequently preventing the transcriptional upregulation of pro-inflammatory cytokines and the cleavage of caspase-1 [49]. Concurrently, glial D2 receptor activation has been shown to induce alphaB-crystallin (CRYAB), a heat-shock protein that physically binds to NF-κB in the cytoplasm, providing an additional layer of inhibition against its nuclear migration [50, 51]. Furthermore, β-arrestin 2 physically associates with the NLRP3 inflammasome core, directly inhibiting its assembly and activation in reactive glia [52]. Through these synergistic mechanisms, D2-like receptor agonists downregulate downstream NF-κB-associated luciferase activity and block p65 nuclear entry [50–52]. Consistent with this, our findings show that rotigotine reduces glial activation and NF-κB/p-p65 signaling in the dorsal horn of 6-OHDA-lesioned rats, which strongly aligns with this neuroprotective and anti-inflammatory mechanism. However, because we did not directly evaluate upstream molecular intermediates such as β-arrestin 2, NLRP3, CRYAB, PPP2R2C, PP2A, and Akt, future investigations will need to fully elucidate the relative contribution of each cascade to rotigotine’s spinally mediated antinociceptive therapeutic effect in our model.

### Unilateral 6-OHDA Lesion of the SNpc Increases Glial Activation, NF-κB/p-p65 Signaling, and the Release of TNF-α and IL-1β in the L5 Spinal Cord Dorsal Horn

Our immunofluorescence data showed that unilateral 6-OHDA lesion of the SNpc increased the number of GFAP^+^ astrocytes, Iba1^+^ microglia, and overall NF-κB/p-p65^+^ immunoreactivity in the ipsilateral L5 dorsal horn, with greater intensity in the superficial laminae I–II. Moreover, consistent with these cellular changes, ELISA analyses confirmed that the unilateral 6-OHDA lesion increased TNF-α and IL-1β levels in the L4–L6 spinal cord. These findings indicate that degeneration of nigrostriatal dopaminergic neurons is associated not only with supraspinal neuroinflammatory changes, but also with a robust spinal glial response in regions critically involved in nociceptive processing.

Previous studies have shown that 6-OHDA lesions trigger a neuroinflammatory response characterized by microglial and astrocytic activation, alongside an increased production of pro-inflammatory mediators [5, 53, 54]. Activated glial cells release cytokines, chemokines, and other inflammatory mediators that amplify neuronal dysfunction and contribute to pain-related sensitization. In this context, the increase in GFAP^+^ and Iba1^+^ cells in the superficial dorsal horn are particularly relevant because laminae I–II are major sites for receiving and integrating nociceptive input from peripheral afferents. Previous reports have shown increased GFAP^+^ cell counts and morphological remodeling of Iba1-labeled cells, such as enlarged somas and retracted, thicker processes, in 6-OHDA-lesioned rats [5]. Furthermore, substantial evidence demonstrates that spinal astrogliosis and microgliosis are pivotal drivers in the development and maintenance of persistent pain states [55–57].

Consistent with these glial changes, we also detected upregulated regional NF-κB/p-p65 (Ser536) immunoreactivity, a marker associated with the p65 signaling pathway. Crucially, our regional Pearson´s correlation analysis revealed a cell-type-specific asymmetry in this response: the NF-κB-p-P65 signal showed a strong positive correlation within astrocytic domains (*r* = 0.6308), in contrast to a negative, uncoupled relationship within microglial profiles (*r* = -0.1975). The NF-κB signaling pathway is a central regulator of inflammatory gene transcription and has been widely implicated in glial activation, synaptic plasticity, and central sensitization in both chronic pain and neurodegenerative conditions [5, 58–61].

In this context, the spatial localization of this upregulation of GFAP^+^ astrocytes and regional NF-κB/p-p65 immunoreactivity within the superficial laminae I-II may have important functional implications for potential pain chronification mechanisms in this model. Laminae I-II constitute a major central gateway from peripheral fibers [62]. Under pathological conditions, the persistent activation of spinal glial cells in these superficial laminae has been suggested to shift their role from acute nociceptive processing to the modulation of chronic pain states [7]. Notably, while microglial activation is frequently associated with the initiation phase of neuroinflammation, reactive astrocytes are often described as important drivers involved in sustaining central sensitization over extended periods [57].

Consequently, our strong positive correlation between NF-κB/p-p65 and astrocytic domains, coupled with the observed increase in downstream pro-inflammatory cytokines, suggests that regional upregulation of the spinal p-65 pathway may contribute to spinal hyperexcitability. While we cannot infer direct cell-specific nuclear activation from these tissue-level observations, activation of the NF-κB pathway, particularly within spinal glial compartments, is known to promote the production of pro-inflammatory cytokines like TNF-α and IL-1β, which sustain spinal neuroinflammation and facilitate nociceptive hypersensitivity [63]. While the spatial resolution constraints of standard epifluorescence microscopy at 10x magnification preclude the verification of direct cell-specific nuclear translocation or functional transcriptional activation, this localized neuroinflammatory response could promote a state of central sensitization, pointing toward a potential neurobiological link between supraspinal nigrostriatal degeneration and spinal neuroinflammation in this model of Parkinson disease.

### Rotigotine Binds to NF-κB/p65 by Molecular Docking

The molecular docking analysis in the present study provides an exploratory computational screening suggesting that rotigotine may interact with the NF-κB complex, particularly within regions corresponding to the p65 subunit. However, this approach is predictive in nature and does not demonstrate direct binding or functional modulation. Rotigotine shows computational evidence of favorable binding energies in both AutoDock 4.2 and AutoDock Vina simulations, with values comparable to CAPE, a well-characterized inhibitor of NF-κB signaling [64]. However, CAPE inhibits NF-κB through covalent modification of cysteine residues in the p50 subunit, a mechanism not captured by the non-covalent docking protocol used here and not reflected in its docked pose, which instead localized to p65. This comparison should therefore be interpreted strictly as a computational benchmarking reference for the docking protocol, not as evidence of an equivalent mechanism of action between CAPE and rotigotine. The differences observed in binding energies may be attributed to the distinct scoring functions and computational parameters employed by each simulator [65].

These findings suggest that rotigotine may have a biologically relevant theoretical binding affinity for NF-κB, supporting the hypothesis that its antinociceptive effects may extend beyond its established role as a dopamine receptor agonist. NF-κB is an important transcription factor involved in the regulation of inflammatory responses and plays a role in the development and maintenance of chronic pain [63]. For this reason, NF-κB signaling is considered an important therapeutic target in neuropathic and inflammatory pain conditions such as PD [5, 61].

Interestingly, docking results identified hydrophobic interactions between rotigotine and Leu280, Gly247, and Pro241 in the p65 region of the NF-κB complex (PDB: 1NFI). These residues are situated near domains involved in protein stability and DNA-binding regulation, suggesting a theoretical site where rotigotine binding could potentially interfere with NF-κB transcriptional activity. While molecular docking alone is insufficient to claim a direct mechanism of action, this computational model offers a speculative mechanistic hypothesis that aligns with the reduction in NF-κB immunoreactivity and downstream cytokines determined in our in vivo experiments following rotigotine administration. Definitive experimental assays, such as biophysical binding studies (e.g., Surface Plasmon Resonance or CETSA) and functional in vitro and in vivo assays, remain strictly necessary to confirm whether rotigotine directly modulates NF-κB.

### Subacute rotigotine Treatment Reduces Glial Activation and NF-κB/p-p65 Signaling in the L5 Spinal Cord Dorsal Horn

In the present study, subacute intranasal treatment with rotigotine (0.5 mg/kg) for five consecutive days significantly reduced the number of GFAP^+^ astrocytes, Iba1^+^ microglia, and NF-κB/p-p65^+^ cells in the L5 dorsal horn, while effectively reversing the upregulation of TNF-α and IL-1β in 6-OHDA-lesioned rats. These findings suggest that rotigotine attenuates spinal gliosis and modulates regional NF-κB/p-p65 signaling within a nociceptive region that is critically involved in the maintenance of hypersensitivity. Remarkably, our spatial correlation analysis showed that rotigotine treatment completely uncoupled the signal association between NF-κB/p-p65 and GFAP^+^ astrocytes, reducing the Pearson´s coefficient to near zero (*r* = 0.0358). Conversely, the treatment showed a robust positive correlation with microglial profiles (*r* = 0.6492), closely resembling the homeostatic pattern observed under the sham condition. This phenotype-specific divergence qualitatively indicates that rotigotine treatment shifts the spatial tissue-level distribution of the p-p65 signal away from astrocytic domains toward microglial cells in the dorsal horn. The marked reduction in astrocytic and microglial activation observed after rotigotine treatment is consistent with the hypothesis that its analgesic effects may be linked, at least in part, to the fine-tuning of neuroinflammatory processes in the spinal cord.

The reduction in glial activation after rotigotine treatment may relate to dopaminergic signaling, particularly through activation of D2/D3 receptors. D2-like receptors are coupled to Gi proteins, leading to inhibition of adenylyl cyclase, reduced intracellular cAMP levels, and subsequent modulation of downstream signaling pathways involved in inflammatory responses [66]. In addition, activation of D2-like receptors has been shown to suppress the production of pro-inflammatory mediators, including TNF-α, IL-1β, and IL-6, partially through inhibition of NF-κB signaling [51]. These observations support the idea that rotigotine may alleviate tactile allodynia and mechanical hyperalgesia by attenuating spinal neuroinflammation through the selective modulation of NF-κB/p-p65 signaling and the subsequent release of proinflammatory cytokines in the spinal dorsal horn.

Finally, several limitations of the present study must be acknowledged when interpreting its translational implications. First, this study was conducted exclusively in male rats. Clinical and preclinical evidence robustly demonstrates that both PD progression and chronic pain processing exhibit significant sexual dimorphism, often modulated by the neuroprotective and antinociceptive actions of estrogenic hormones. Therefore, our findings regarding intranasal rotigotine efficacy and its underlying spinal glial mechanisms cannot be directly generalized to female populations without further targeted validation. Second, our subacute treatment protocol spanned a relatively short period of five consecutive days. While this timeline was sufficient to demonstrate a robust reversal of established tactile allodynia and mechanical hyperalgesia alongside a suppression of the spinal NF-κB pathway, it represents a sub-acute therapeutic window. Because PD is a chronic, progressive neurodegenerative disorder, future long-term studies are strictly required to evaluate potential drug tolerance, cumulative toxicity, or sustained disease-modifying effects. Consequently, while our results provide a strong mechanistic foundation for targeting D2/D3 receptors via the intranasal route, we must interpret these translational implications cautiously before extrapolating them to complex human clinical settings.

In conclusion, these data indicate that unilateral 6-OHDA lesion of the SNpc correlates with changes in pain behavior and spinal neuroinflammatory markers, including glial activation, L5 dorsal horn NF-κB/p-p65 immunoreactivity, and L4-L6 spinal cytokine levels. Acute and subacute intranasal rotigotine treatment reduced mechanical hypersensitivity, a response our antagonist experiments suggest may be modulated in part by D2 and D3 receptors. Subacute administration also coincided with reduced astrocytic and microglial activation, NF-κB/p-p65 immunoreactivity, and spinal cytokines. Collectively, these observations support a potential link between rotigotine’s anti-hypersensitivity effects and modulation of spinal neuroinflammation in these models. Nonetheless, further research is required to elucidate the precise mechanisms and determine their relevance to pain in PD. Lastly, the molecular docking data provide a preliminary hypothesis, and direct binding between rotigotine and NF-κB requires experimental confirmation.

## Data Availability

The datasets generated and/or analyzed during the current study are not publicly available due to institutional and licensing restrictions. However, they are available from the corresponding author upon reasonable request.
